# Daily rhythm of salivary and serum urea concentration in sheep

**DOI:** 10.1186/1740-3391-4-16

**Published:** 2006-11-23

**Authors:** Giuseppe Piccione, Augusto Foà, Cristiano Bertolucci, Giovanni Caola

**Affiliations:** 1 Dipartimento di Morfologia, Biochimica, Fisiologia e Produzione Animale, Laboratorio di Cronofisiologia Veterinaria, Facoltà di Medicina Veterinaria, Università di Messina, 98168 Messina, Italy; 2 Dipartimento di Biologia ed Evoluzione, Università di Ferrara, via L. Borsari 46, 44100 Ferrara, Italy

## Abstract

**Background:**

In domestic animals many biochemical and physiological processes exhibit daily rhythmicity. The aim of the present study was to investigate the rhythmic pattern of salivary and serum urea concentrations in sheep.

**Methods:**

Six 3-year-old female sheep kept in the same environmental conditions were used. Sheep were sampled at 4 hour intervals for 48 consecutive hours starting at 08:00 of the first day and finishing at 04:00 of the second day. Blood samples were collected via intravenous cannulae inserted into the jugular vein; saliva samples were collected through a specific tube, the "Salivette". Salivary and serum urea concentrations were assayed by means of UV spectrophotometer. ANOVA was used to determine significant differences. The single Cosinor procedure was applied to the results showing significant differences over time.

**Results:**

ANOVA showed a significant effect of time on salivary and serum urea concentrations. Serum and salivary urea peaked during the light phase. In the dark phase serum and salivary urea concentrations decreased, and the diurnal trough occurred at midnight. Cosinor analysis showed diurnal acrophases for salivary and serum urea concentrations. Daily mean levels were significantly higher in the serum than in the saliva.

**Conclusion:**

In sheep both salivary and serum urea concentrations showed daily fluctuations. Urea is synthesized in the liver and its production is strongly influenced by food intake. Future investigation should clarify whether daily urea rhythms in sheep are endogenous or are simply the result of the temporal administration of food.

## Background

The circadian clock, an endogenous timing system, generates biochemical, physiological and behavioural rhythms. To be useful, these clocks must be synchronized (*entrained*) to environmental time cues (*zeitgebers*). The primary environmental zeitgeber is light, and the regular daily change in light intensity at dawn or dusk seems to determine the circadian photo entrainment. Circadian rhythms have been described in many animal species, including livestock [1,2]. Some molecular studies on rodents identified the liver as the site of a putative food-entrainable oscillator [3], which could be synchronized by feeding time [4,5]. Few studies were carried out on the rhythmicity of liver function in farm animals. Serum concentration of urea was evaluated in cows during different feeding schedules [6] and in goats maintained under various schedules of lighting and feeding [7] in order to understand the mechanisms of entrainment of liver function. Ruminants, such as sheep and cattle, secrete a large amount of saliva from the salivary glands into the rumen (≥100 litre/day in cattle and ≥10 litre/day in sheep). In ruminants, the recycling of urea to the fore stomach is an importance source of nitrogen for synthesis of microbial protein [8,9]. For a given diet, the amount of urea recycled to the rumen, both in saliva and across the rumen wall, is directly related to the amount of urea synthesized which, in turn, is related to nitrogen intake and the degradability of dietary nitrogen. This explains how serum urea concentration is strictly related to feeding: when a nitrogen-deficient ration is ingested, urea does not pass into the urine but is converted into microbial protein in the digestive tract, to be re-utilized [10]. Both salivary secretion and direct diffusion through the rumen wall are responsible for the appearance of serum urea in the digestive tract. In sheep, a high correlation between urea concentration in parotid saliva and in plasma was also observed [11]. The defining of the liver as a site of a putative food-entrainable oscillator and the existence of a daily rhythm of serum urea concentration influenced by feeding in ruminants led us to investigate the rhythmic pattern of both salivary and serum urea concentrations in sheep.

## Methods

Six 3-years-old female sheep (*Ovis aries*, Comisana breed; mean body weight 48.0 ± 2.0 kg) clinically healthy, non-pregnant and non-lactating, were used. Animals were housed in individual boxes and kept under natural photo-thermoperiodic conditions (longitude: 15° 33' 24" E, latitude: 38° 12' 27" N; sunrise: 05:52, sunset: 17:43). Starting 30 days before the test, all sheep were fed with hay *ad libitum *and concentrate 250 g/day (oats 25%, corn 34%, mineral vitamin supplement 3% and barley 38%) once each day at 07:00 h. Water was available *ad libitum*. After this preconditioning period, saliva and serum samples were collected every 4 hours for two consecutive days (starting at 08:00). Protocols of animal husbandry and experimentation followed applicable regulations in Italy. Salivary samples were collected through a specific tube, the "Salivette^®^"(SARSTEDT, Germany), which provides a standardized method for the easy and safe collection of saliva. Briefly, the "salivette" is a tube containing a swab used to absorb the saliva. The swab was attached to a nylon thread and inserted in the mouth. Sheep were stimulated to chew for 1–2 minutes to fill the swab with as much saliva as possible. To recover a saliva sample (0.5–1.5 ml) from the swab, the salivette was centrifuged at 2000 × g for 2 minutes. The swab was removed from the salivette and the saliva collected in the tube for analysis. Saliva obtained was immediately stored at -20°C until assayed. Blood samples (5 ml) were collected using jugular intravenous catheters (FEP 20 g 1 × 32 mm; Delta Med, Italy) into tubes *Vacuitainer *without anticoagulant. Blood samples clotted at room temperature for 1 h and were subsequently centrifuged at 3000 × g for 20 min (4235 A, ALC, Italy). The obtained sera were stored at -20°C until assayed. Salivary and serum urea was analyzed with a standard kit (SEAC, Italy) by means of a UV spectrophotometer (SEAC, Italy). The urea kit is based on the breakdown of urea into ammonia and CO_2 _by the action of urease followed by the synthesis of glutamate and NAD+ by the reaction of ammonia, α-chetoglutarate and nicotinamide adenindinucleotide. All the results were expressed as mean ± SD. Data were normally distributed (*p *< 0.05, Kolmogorov-Smirnov test) and one-way or two-way repeated measures analysis of variance (ANOVA) was used to determine significant differences (*p *values < 0.05 were considered statistically significant). Bonferroni's Multiple Comparison test was applied for post hoc comparison. To compare overall levels of urea in the different analyses, mean urea levels over a daily period were used. Data were analyzed using the software STATISTICA 5.5 (StatSoft Inc., USA). In addition, we applied a trigonometric statistical model to the average values of each time series, so as to describe the periodic phenomenon analytically, by individuating the main rhythmic parameters according to the single cosinor procedure [12]: Mesor (Midline Estimating Statistic of Rhythm), expressed in the same conventional unit of the relative parameter, with the confidence interval (C.I.) at 95%, Amplitude (A), expressed in the same unit as the relative Mesor, and Acrophase (Φ), expressed in hours with 95% confidence intervals.

## Results and Discussion

**Figure 1:**
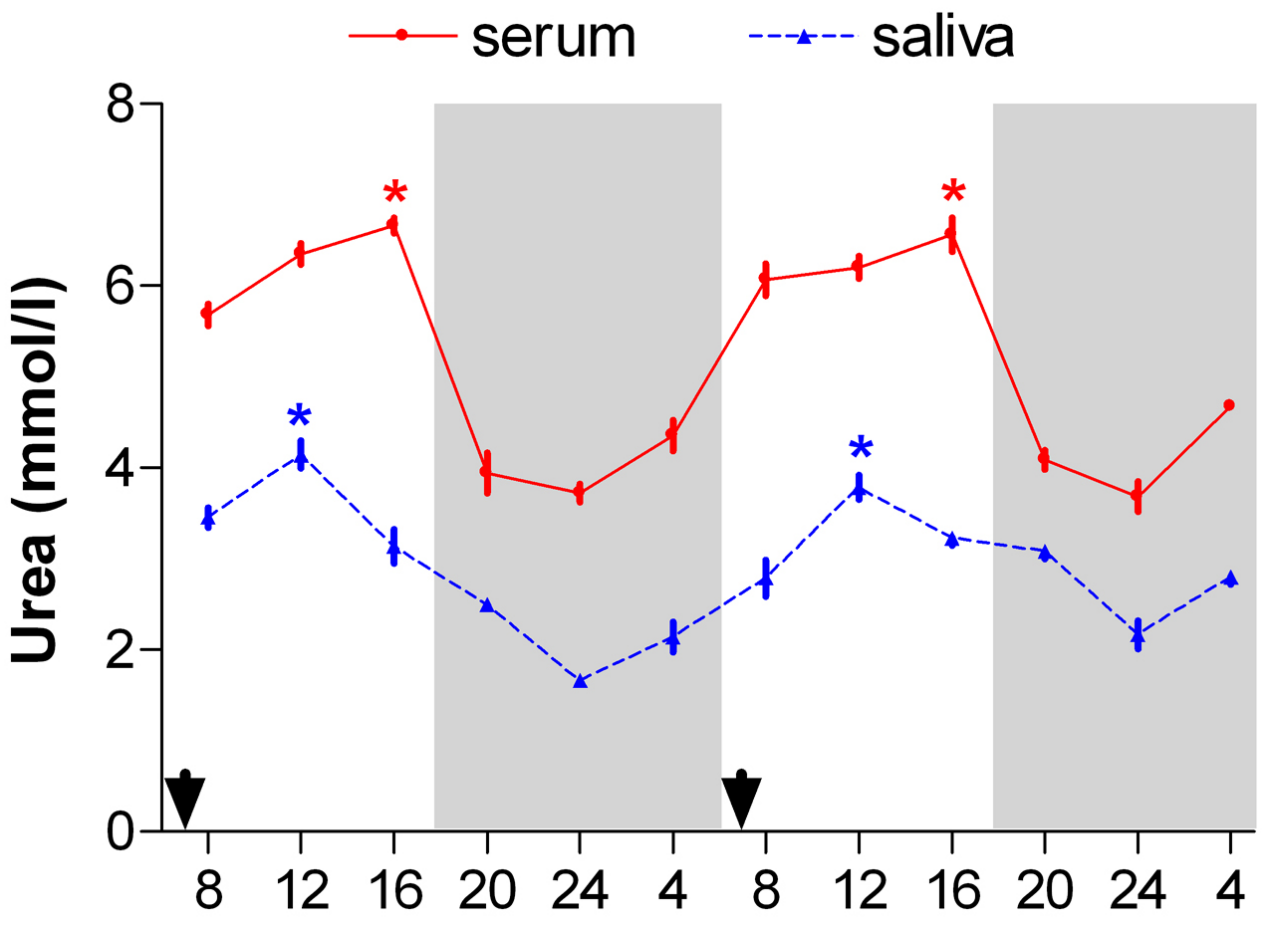
**Daily rhythms of serum and salivary urea concentrations in sheep**. Both urea profiles showed clear diurnal rhythms: urea levels were high during the light phase and low during the dark phase of the natural light-dark cycle. Each point represents mean ± SEM. Gray bars indicate the dark phase of the natural light-dark cycle. Arrowheads indicate times of feeding. Asterisks indicate peaks of urea concentrations.

ANOVA showed a robust daily rhythm of urea in serum and saliva of sheep (serum: F_(11,55) _= 69.64, *p *< 0.0001; saliva: F_(11,55) _= 30.25, *p *< 0.0001; one-way ANOVA). Both urea profiles showed high levels during light phases and low during dark phases (Figure 1). The application of the periodic model and a statistical analysis of the cosinor enabled us to define the periodic parameters and their acrophases (expressed in hours) during the 2 days of monitoring. Table 1 shows the MESOR, with the fiducial limits at 95%; the amplitude, expressed in the same unit as the relative MESOR; the acrophase, calculated using the single cosinor method and expressed in hours, together with the confidence interval at 95%, for the periodic serum and salivary urea concentrations. Serum and salivary urea showed similar diurnal acrophases: serum urea at 12.24 (day 1) and at 11.56 (day 2), salivary urea at 12:00 (day 1) and at 12:12 (day 2).

**Table 1:** Mesor (M), fiducial limits (F.L.) at 95%, Amplitude (A) and Acrophase (Φ), expressed in hours, with confidence interval (C.I.) at 95%, of serum and salivary urea during the two days of study

*Serum urea*					
	
	**MESOR**	**F.L. 95%**	**A**	Φ	**C. I. 95%**
Day 1	5.12	(4.56–5.67)	1.56	12:24	(08:16–16:32)
Day 2	5.21	(4.66–5.76)	1.48	11:56	(07:36–16:16)
*Salivary urea*					
Day 1	2.84	(2.59–3.09)	1.16	12:00	(09:44–14:16)
Day 2	2.75	(2.49–3.01)	0.72	12:12	(08:04–16:20)

Serum urea values were significantly different from salivary values (F_(1,66) _= 464.8, *p *< 0.0001; two-way ANOVA). Particularly, daily mean levels were significantly higher in the serum (5.17 ± 0.15 mmol/l; mean ± SEM) than in the saliva (2.9 ± 0.09 mmol/l).

Our results are comparable to those of a previous study on sheep fed twice a day, in which serum urea levels showed diurnal acrophases at 16:00 [13]. The small difference in acrophases may be explained by differences in the feeding regimes. In fact, investigations in small ruminants have shown that serum urea concentration exhibited daily fluctuations only in the presence of a daily feeding regime: a robust daily rhythm was observed in goats fed once each day, which vanished when animals were food deprived [7].

Our results cannot exclude the possibility that the increase of urea from 04:00 to 08:00 was due to the start of feeding at 07:00. Other investigations clearly showed a circadian rhythm of urea with diurnal peaks in cows [14] and documented the effect of different feeding schedules on daily rhythms of serum urea and ammonia concentration [6,15]. Monogastric animals also showed a daily rhythm of plasma urea concentration with diurnal acrophases [13]. For instance, peaks of plasma urea concentration were reached 4 hours after feeding in pigs fed twice each day compared to subjects fed *ad libitum *[16].

## Conclusion

Here we showed a non-invasive method to measure daily variations of urea concentrations. However, we must consider that urea levels in the saliva are significantly lower than in the serum. Serum and salivary urea are synthesized in the liver and their production is strongly influenced by food intake. Our results suggest the influence of external stimuli (feeding time) on the rhythmic pattern of metabolites involved in liver function, possibly acting on circadian clocks in the liver and the suprachiasmatic nucleus, which could be very important for the ability of organisms to synchronize their internal physiology. Future investigation should clarify whether daily urea rhythms in sheep are endogenous or are simply the result of the temporal administration of food.

## Competing interests

The author(s) declare that they have no competing interest.

## Authors' contributions

GP directed the study, participated in data collection and wrote the final version of the manuscript. AF participated in the design of the study. CB participated in the design of the study and performed statistical analysis. GC participated in the design of the study and helped with its coordination. All authors read and approved the final version of the article.
